# KLF4 orchestrates a Ccl2+ fibroblast-mediated inflammatory network driving preterm birth

**DOI:** 10.3389/fmed.2025.1700275

**Published:** 2026-01-30

**Authors:** Jing Gao, Yaqing Chang, Yuan Su, Lulu Sun, Fang Wang, Hong Xin

**Affiliations:** 1 Department of Obstetrics, The Fourth Hospital of Shijiazhuang, Shijiazhuang, Hebei, China; 2 Department of Obstetrics, The Second Hospital of Hebei Medical University, Shijiazhuang, Hebei, China

**Keywords:** fibroblast, inflammation, KLF4, Mendelian randomization, preterm birth

## Abstract

**Background:**

Preterm birth (PTB) remains the leading cause of neonatal mortality and long-term complications, yet its molecular mechanisms are incompletely understood. Fibroblasts, as critical components of the gestational microenvironment, have an undefined role in PTB pathogenesis.

**Methods:**

This study employed single-cell RNA sequencing (scRNA-seq) datasets from murine preterm birth models (GSE200289 and E-MTAB-11491), processed using Seurat and Harmony for clustering and integration, to analyze cellular heterogeneity. This was integrated with Mendelian randomization (MR) using eQTLs from FinnGen and IEU GWAS databases as instrumental variables, and validated through functional experiments including LPS-induced placental explant cultures, qRT-PCR, western blotting, immunohistochemistry, and an *in vivo* rat intrauterine infection model with AAV-shKLF4 knockdown.

**Results:**

We first established a single-cell transcriptomic atlas of preterm mice, identifying nine cell clusters, among which the Ccl2+ fibroblast subpopulation (Ccl2+fib) exhibited significant expansion under pathological conditions. MR analysis identified KLF4 as a genetic risk factor for PTB (OR = 1.32, 95% CI: 1.12–1.56), with reverse MR confirming the absence of reverse causality. Clinical validation revealed KLF4-specific upregulation in peripheral blood and placental tissues of PTB patients. Functional studies demonstrated dose-dependent KLF4 upregulation in LPS-induced placental inflammation, while KLF4 knockdown suppressed TNF-α and IL-1β secretion and reduced apoptosis. In a rat intrauterine infection model, KLF4 knockdown decreased LPS-induced preterm birth rates from 65% to 20%, accompanied by significantly reduced fetal stillbirth rates.

**Conclusion:**

This study reveals that the fibroblast subpopulation Ccl2+fib contributes to preterm birth through a KLF4-dependent inflammatory regulatory network. KLF4 downregulation significantly reduces infection-induced preterm birth rates and adverse pregnancy outcomes, suggesting that KLF4 is a critical therapeutic target for preterm birth.

## Introduction

Preterm birth (PTB) remains one of the most pressing challenges in obstetrics and neonatology and is a leading cause of neonatal mortality and long-term morbidity worldwide (1). Although survival of preterm infants has improved with advances in neonatal intensive care, the burden of PTB-related complications—including bronchopulmonary dysplasia, necrotizing enterocolitis, and neurodevelopmental impairment—remains substantial (2). Importantly, PTB is etiologically heterogeneous and influenced by multiple risk factors spanning reproductive tract conditions, maternal comorbidities, immune dysregulation, genetic susceptibility, and obstetric complications (3–5). This complexity has limited the development of effective preventive strategies and targeted therapies.

A growing body of evidence highlights inflammation at the maternal–fetal interface as a central pathway that can precipitate PTB, particularly in infection-associated contexts where dysregulated innate and adaptive immune responses may prematurely activate parturition-related programs (6, 7). However, the inflammatory response during pregnancy is not driven by immune cells alone. The maternal–fetal interface is a tightly orchestrated microenvironment in which stromal cells and immune cells communicate to maintain tissue integrity, vascular adaptation, and immune tolerance. Fibroblasts, as key stromal components, can actively shape local immunity through cytokine/chemokine production and cell–cell communication networks. Yet fibroblasts are highly heterogeneous, and the specific fibroblast states that expand during PTB and the regulatory programs that govern their inflammatory behavior remain insufficiently defined.

Krüppel-like factor 4 (KLF4) is an evolutionarily conserved transcription factor implicated in cellular differentiation, proliferation, apoptosis, and stress responses (8–10). In inflammatory diseases, KLF4 has been reported to modulate immune and metabolic homeostasis and to participate in cytokine-associated signaling programs in a context-dependent manner (11–13). Emerging evidence further suggests that KLF4 is relevant to reproductive biology, including trophoblast function and placental vascular or angiogenic regulation, supporting a potential role in maintaining maternal–fetal homeostasis (14). Nevertheless, a direct and coherent mechanistic framework linking KLF4 to PTB—especially through defined cellular contexts at the maternal–fetal interface—has been limited. This gap motivated us to evaluate whether KLF4 is associated with PTB risk and whether it may participate in stromal-driven inflammatory amplification.

To address these questions, we leveraged complementary approaches that connect cellular resolution, genetic inference, and functional validation. Single-cell RNA sequencing (scRNA-seq) enables high-resolution dissection of cellular heterogeneity and state transitions within complex tissues (15), while Mendelian randomization (MR) provides an instrumental-variable framework to support causal inference and prioritize candidate regulators beyond observational association (16). Finally, to model infection-associated inflammatory stress relevant to PTB, we employed lipopolysaccharide (LPS)-based experimental systems. LPS is a canonical pathogen-associated molecular pattern that activates innate immune signaling cascades and induces robust pro-inflammatory responses, making it a widely used paradigm for investigating infection-triggered mechanisms implicated in PTB (14).

In this study, we integrate scRNA-seq profiling of a PTB model, MR-based prioritization, clinical observations, and *in vivo*/*in vitro* functional perturbation to investigate fibroblast heterogeneity and to evaluate KLF4 as a candidate regulatory node. By linking an expanded inflammatory fibroblast state (Ccl2^+^ fibroblasts) to genetically supported risk association and functional outcomes under inflammatory challenge, we aim to provide a coherent cellular–genetic–functional framework for inflammation-associated PTB and to inform future translational studies targeting key regulatory pathways at the maternal–fetal interface.

## Materials and methods

### Data preparation and processing

Single-cell RNA sequencing datasets related to murine preterm birth were obtained from Gene Expression Omnibus (GSE200289) and ArrayExpress (E-MTAB-11491). Data processing included quality control and normalization. Sample-specific filtering thresholds were calculated based on UMI counts, gene counts, and mitochondrial gene percentages (pctMT). Cells were filtered using the following criteria: minGene = 200, maxGene = 5,000, and pctMT = 10. High-quality gene expression data were normalized, and 2,000 highly variable genes common across all samples were identified for downstream analysis. Principal component analysis (PCA) was performed on scaled data to reduce dimensionality. To mitigate technical batch effects arising from different sample origins, Harmony integration was implemented using the orig.ident variable as the batch covariate. This was performed within the RNA assay space over 50 iterations. The top 30 principal components (PCs) were computed and subsequently integrated using Harmony. For non-linear dimensionality reduction and visualization, uniform manifold approximation and projection (UMAP) was applied to the harmony-corrected components, utilizing an increased neighborhood size (*n* = 300) to enhance local structure preservation. A k-nearest neighbor graph was constructed based on this integrated space, and cell clustering was performed using the Leiden algorithm at a resolution of 0.1, yielding an initial partition of 12 clusters.

### Fibroblast subpopulation identification

A total of 30,451 fibroblasts were further clustered at resolution = 0.2, the subpopulations were named after their top marker genes (Atp8a1, Lum, Ccl2, Fos, Prap1, Il1b), identified based on stringent criteria (logFC ≥ 1.5, adjusted *p* ≤ 0.05).

### Cell-cell communication analysis

Putative doublets were identified and removed using DoubletFinder prior to downstream analysis. Significant communication pathways and ligand–receptor interactions were identified using CellChat (v1.1.3) with a Benjamini–Hochberg-adjusted *P* < 0.05. Pathway importance was ranked using netAnalysis_signalingRole and rankNet, and representative ligand–receptor pairs were extracted with extractEnrichedLR functions. Full results are provided in Supplementary Table 1.

### Mechanistic exploration of Ccl2+fib subpopulation

Differentially expressed genes (DEGs) between Ccl2+fib and other subpopulations (Atp8a1+fib, Lum+fib, etc.) were identified. Instrument strength and pleiotropy were evaluated using F-statistics and MR-Egger intercept tests, and Instrumental variables (IVs) were selected from eQTLs of key marker genes (FinnGen and IEU GWAS databases, *p* < 5e-08, FDR > 10). Mendelian randomization (MR) analyses estimated odds ratios (ORs) for PTB, followed by reverse MR to exclude reverse causality.

### Pseudotime trajectory analysis

Pseudotime trajectory analysis was performed using both Monocle 3 to ensure methodological robustness. From the annotated Seurat object, the scaled count matrix of highly variable genes and UMAP coordinates were extracted. Raw count data were normalized using size factors derived from trajectory analysis.

### Clinical specimens

Placental tissues were collected from 84 parturients (50 term, 34 preterm) at The Fourth Hospital of Shijiazhuang. Eligibility was confirmed by standardized antenatal examinations and clinical records. Inclusion criteria: (1) Complete antenatal care and delivery at our institution. (2) Signed informed consent. (3) No preoperative treatments. Exclusion criteria: (1) History of cervical conization, uterine anomalies, chorioamnionitis, or intrauterine infection. (2) Recurrent vaginal bleeding. (3) Severe organ dysfunction. (4) Fetal congenital malformations. This study has been approved by the Ethics Committee of the Fourth Hospital of Shijiazhuang (No. 20250034).

### LPS-induced preterm rat model

The LPS dose and timing were selected based on prior study and pilot optimization (17). Time-pregnant Sprague–Dawley (SD) rats received intrauterine injections of LPS (416 μg/kg) or PBS (control) under isoflurane anesthesia on gestational day (GD) 17.

### KLF4 overexpression animal model

At 1 h post-LPS injection (GD17), rats were randomized to receive tail vein injections of AAV-shKLF4 (74 nmol/kg) or AAV-shNC (100 μL, intraperitoneal). Groups: LPS + AAV-shKLF4, AAV-shKLF4, LPS + AAV-shNC, and AAV-shNC. SD rats were purchased from the Laboratory Animal Center of Hebei Medical University. All animal experiments were approved by the Laboratory Animal Ethics Committee of the Fourth Hospital of Shijiazhuang and conducted strictly in accordance with the Chinese Guidelines for the Ethical Review of Laboratory Animal Welfare to ensure full protection of animal welfare.

### Placental explant culture

Placentas from GD17 SD rats were aseptically dissected, rinsed in saline, and halved. Explants were cultured in DMEM/F12 medium with LPS or AAV-shKLF4 (2 mL/well) at 37 °C (5% CO_2_, 90% humidity) for 24 h.

#### RNA extraction and quantitative real-time PCR (qRT-PCR)

Placental explants: Tissues were collected after 24-h treatments (LPS gradient or AAV-shKLF4). Rat placental tissues: Placenta from LPS-induced intrauterine infection models (LPS group) and shKLF4-treated rats (LPS + shKLF4 group) were harvested at gestational day 18. Total RNA was extracted using TRIzol reagent (Invitrogen) according to the manufacturer’s protocol. Reverse transcription was performed with 1 μg RNA using the PrimeScript RT Reagent Kit (Takara). Reactions were carried out in triplicate using SYBR Green Master Mix (Roche) on a LightCycler 480 system (Roche). Cycling conditions: 95 °C for 30 s, followed by 40 cycles of 95 °C for 5 s and 60 °C for 30 s. Primer sequences:

TNF-α: F 5′-GCTGCTTCCAAACCTTTGAC-3′, R 5′-AGCTTCTCCACAGCCACAAT-3′
IL-1β: F 5′-TCCAGCTGTAGAGTGGGCTT-3′, R 5′-GCTGAGGAAGATGCTGGTTC-3′
IL-6: F 5’-AGTTGTGCAATGGCAATTCTGA-3’, R 5’-CTCTGGCTTTGTCTTTCTTGTTATCTTT-3’
Relative mRNA levels were calculated using the 2^–ΔΔCt^ method normalized to GAPDH.

#### Western blot analysis

Placental explants: Tissues were lysed in RIPA buffer (Beyotime) containing protease inhibitors (Roche). Rat placental tissues: Homogenized in ice-cold lysis buffer and centrifuged at 12,000×*g* for 15 min at 4 °C. Protein concentrations were quantified via BCA assay (Pierce). 30 μg protein per sample was separated by 10% SDS-PAGE and transferred to PVDF membranes (Millipore) using a semi-dry transfer apparatus (Bio-Rad). Membranes were blocked with 5% non-fat milk in TBST for 1 h, then incubated overnight at 4 °C with primary antibodies: Anti-KLF4 (1:1,000, Abcam, ab215036),Anti-β-actin (1:5,000, Proteintech, 66009-1-Ig). HRP-conjugated secondary antibodies (1:5,000, Cell Signaling Technology) were applied for 1 h at room temperature. Bands were visualized using ECL substrate (Millipore) and quantified via ImageJ software. β-actin served as the loading control.

### Immunohistochemistry (IHC)

Placental explants and rat placental tissues were fixed in 4% paraformaldehyde (PFA) for 24 h at 4 °C, dehydrated through a graded ethanol series, and embedded in paraffin. Sections (4 μm thickness) were cut using a microtome (Leica, Germany) and mounted on poly-L-lysine-coated slides. Slides were deparaffinized in xylene and rehydrated through descending ethanol concentrations. Antigen retrieval was performed by heating sections in 10 mM sodium citrate buffer (pH 6.0) at 95 °C for 20 min using a microwave. Endogenous peroxidase activity was quenched with 3% hydrogen peroxide for 10 min at room temperature. Sections were blocked with 5% normal goat serum (Vector Laboratories, USA) in PBS for 1 h. Anti-KLF4 (1:200, Abcam, ab215036) or anti-cleaved caspase-3 (1:500, Cell Signaling Technology, #9664) was applied overnight at 4 °C. After washing, sections were incubated with HRP-conjugated secondary antibody (1:500, Dako, Denmark) for 1 h at 37 °C. Diaminobenzidine (DAB, Dako) was used as the chromogen for 3–5 min. Nuclei were counterstained with hematoxylin for 1 min. Slides were dehydrated, cleared in xylene, and mounted with neutral balsam. Images were captured using a light microscope (Nikon Eclipse Ci, Japan) equipped with a digital camera (Nikon DS-Ri2). KLF4 expression was evaluated by calculating the percentage of positively stained cells in five randomly selected fields per section using ImageJ software.

#### Statistical analysis

All data were analyzed using the SPSS 21.0 statistical software program (IBM Corporation, Armonk, NY, USA). Graphs were generated with GraphPad Prism 8.0 Software (GraphPad Software, Inc., San Diego, CA). Student’s *t*-tests were used. For *t*-tests, a two-tailed *p* < 0.05 was required for results to be considered as statistically significant.

## Results

### Single-cell transcriptomics reveals the cellular landscape of preterm birth

**FIGURE 1:**
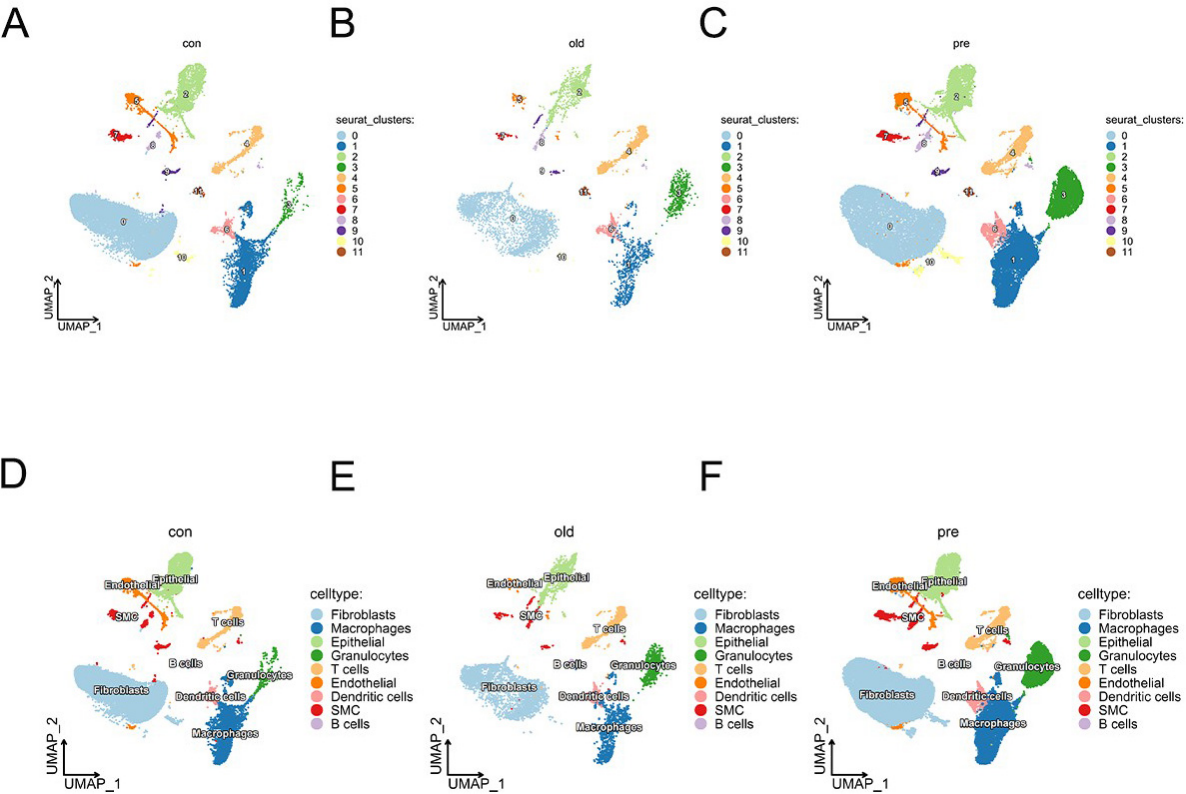
Single-cell RNA sequencing identifies nine distinct cell clusters in preterm birth model mice. **(A–C)** Integrated clustering post-quality filtering identifies 12 clusters in preterm samples. **(D–F)** Subclustering highlights refined subpopulation diversity, with fibroblasts showing the greatest expansion under pathological conditions.

Following unsupervised clustering, cell populations were annotated based on the expression of canonical lineage marker genes. Differentially expressed genes for each of the 12 initial clusters were identified using the FindAllMarkers function in Seurat. Cluster identity was assigned by cross-referencing these differential expression profiles with established cell-type-specific markers. The key marker genes employed for annotation were as follows: T cells (Cd3e, Cd3g, Ptprc); B cells (Cd19, Cd79a, Ms4a1); Smooth Muscle Cells (SMC) (Myh11, Mylk, Actg2); Epithelial cells (Epcam, Krt19); Fibroblasts (Fap, Col1a1, Col3a1, Dcn, Acta2); Endothelial cells (Cldn5, Cdh5); Granulocytes (S100a8, S100a9, Csf3r); Macrophages (Cd68, Cd163, Cd14, Lyz); and Dendritic cells (Clec9a, Xcr1, Batf3) (Figures 1A–C). During the annotation process, clusters that co-expressed marker genes from two or more distinct lineages were identified as potential doublets and removed from subsequent analysis. Furthermore, three initial sub-clusters displayed highly similar SMC marker profiles (Myh11, Mylk, Actg2) without evidence of distinct functional identities at this broad annotation level. Therefore, these three sub-clusters (7–9) were consolidated into a single, broader “SMC” population (Figures 1D–F). This refinement process—involving doublet removal and the merging of transcriptionally similar fibroblast subgroups—resulted in the final annotation of 9 major cell populations.

### Fibroblast subclustering reveals six functionally distinct subpopulations

**FIGURE 2:**
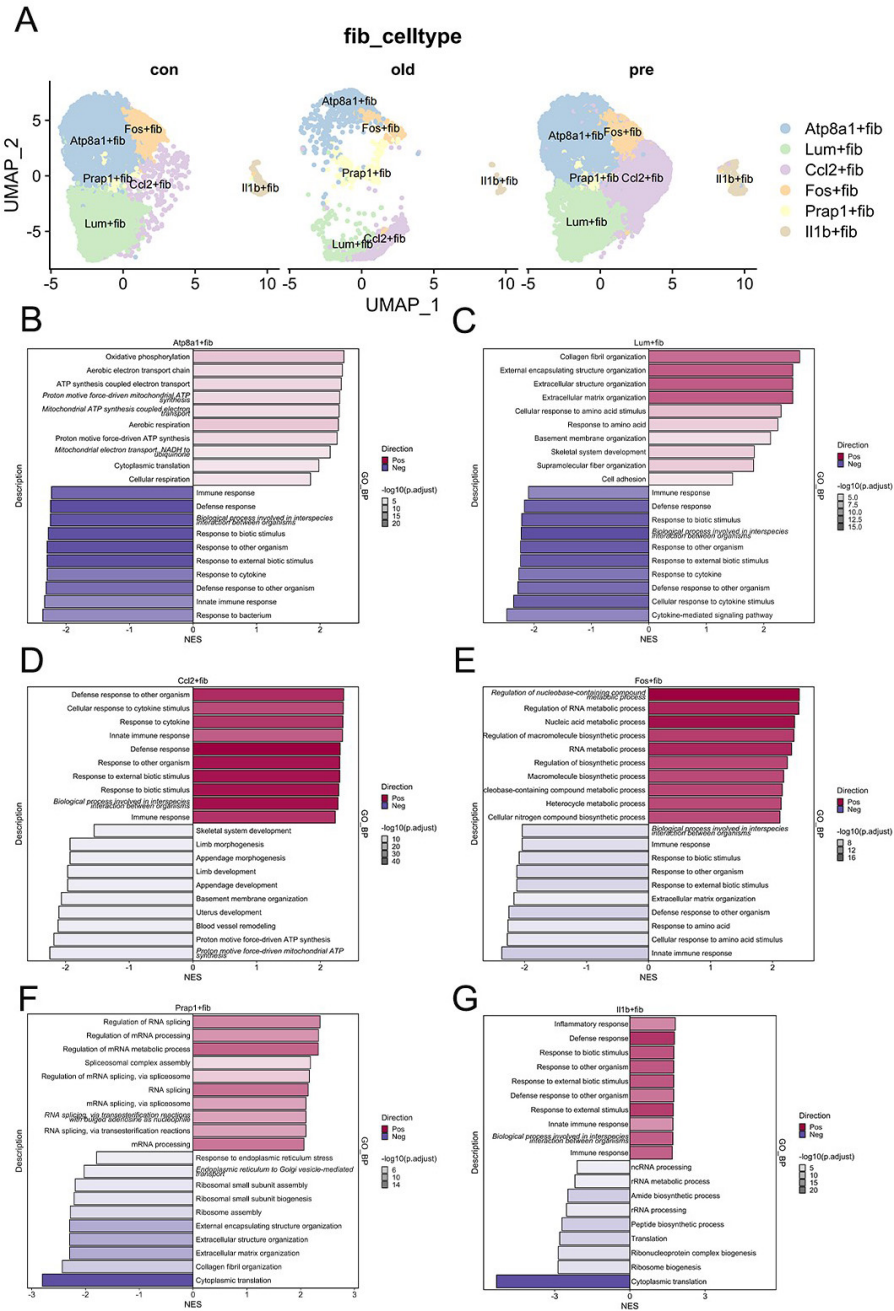
Fibroblast subclustering reveals six functionally distinct subpopulations. **(A)** UMAP visualization shows Atp8a1+fib, Lum+fib, Ccl2+fib, Fos+fib, Prap1+fib, and Il1b+fib clusters. **(B–G)** Enrichment analyses reveal energy metabolism dominance in Atp8a1+fib, immune activation in Lum+fib, reduced vascular remodeling in Ccl2+fib, metabolic processes in Fos+fib, RNA regulation in Prap1+fib, and pro-inflammatory pathways in Il1b+fib.

**FIGURE 3:**
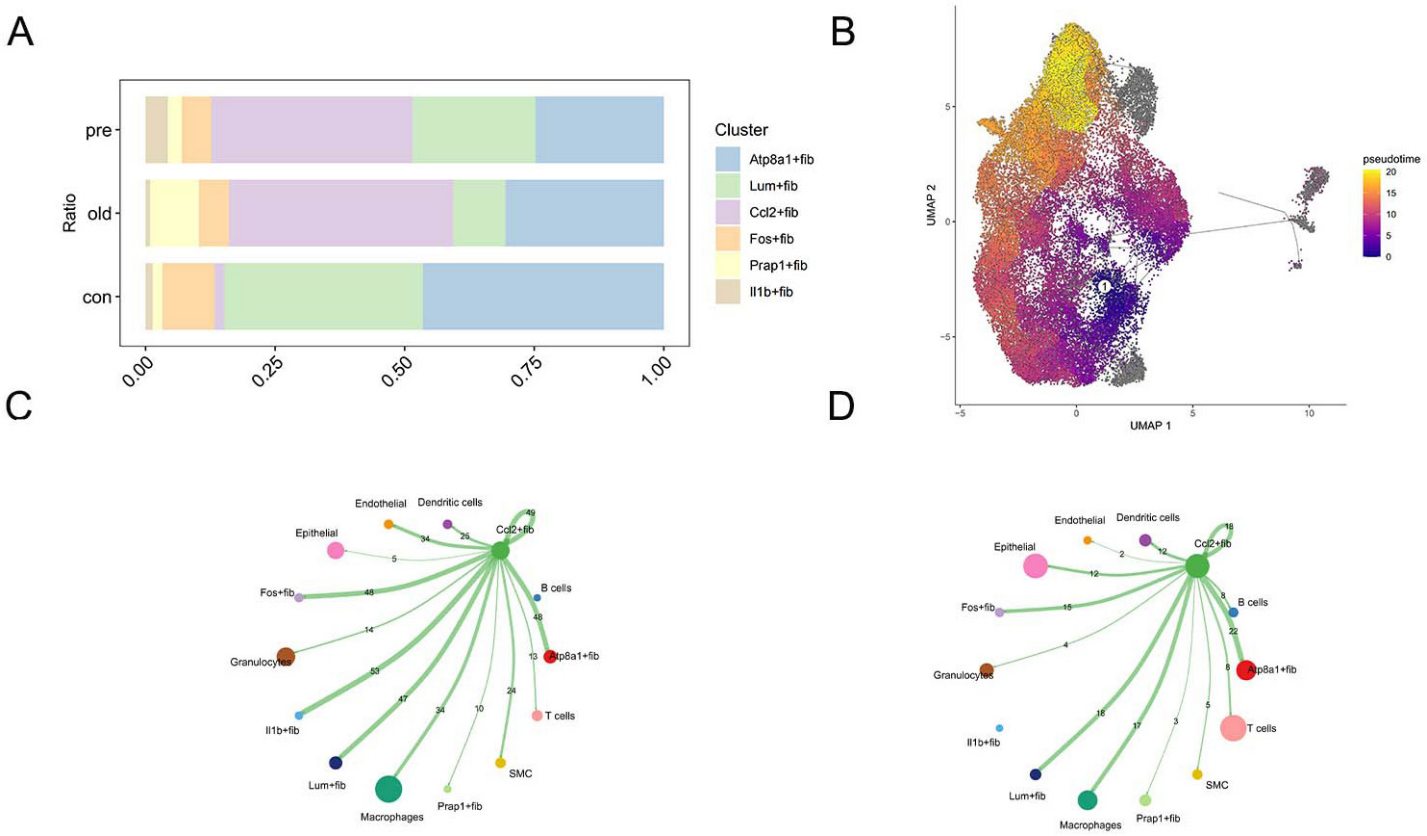
Ccl2+fib dominates fibroblast subpopulations. **(A,B)** Proportional changes of fibroblast subpopulations, with Ccl2+fib significantly elevated in disease groups. **(C,D)** Cell-cell communication analysis reveals the dominant role of Ccl2+fib in intercellular signaling.

Given fibroblasts’ emerging significance in immunological contexts and their understudied role in preterm birth, we focused on fibroblast heterogeneity. Following extraction of 30,451 fibroblasts, subclustering at resolution = 0.2 identified six distinct subpopulations (Figure 2A): Atp8a1+fib, Lum+fib, Ccl2+fib, Fos+fib, Prap1+fib, and Il1b+fib, named according to their top differentially expressed genes (logFC ≥ 1.5, adjusted *p* ≤ 0.05) and literature evidence. Functional enrichment analysis revealed subpopulation-specific signatures (Figures 2B–G), Atp8a1+fib enriched in energy metabolism pathways with attenuated inflammatory responses. Lum+fib dominated defense responses and immune activation. Ccl2+fib exhibited reduced energy regulation, uterine development, and vascular remodeling capacity despite partial overlap with Lum+fib functions. Fos+fib associated with biosynthesis and metabolic processes. Prap1+fib linked to RNA splicing and translational regulation. Il1b+fib demonstrated pronounced pro-inflammatory activity. Notably, Ccl2+fib showed significant expansion across disease cohorts (Figures 3A, B), suggesting its pathogenic potential in PTB. CellChat analysis further revealed Ccl2+fib as a dominant signaling hub in both young and aged cohorts (Figures 3C, D), with enhanced outgoing/incoming communication probabilities. At the ligand–receptor level, we identified representative interactions supporting the above pathway signals. Specifically, Ccl2^+^ fibroblasts contributed key chemokine-mediated interactions (e.g., CCL2–CCR2), and additional inflammatory ligand–receptor pairs were enriched between fibroblasts and immune cells (adjusted *P* < 0.05). A full ranked list of significant ligand–receptor pairs, is provided in Supplementary Table 1.

### Mendelian randomization supports KLF4 as a genetic risk factor for preterm birth

**FIGURE 4:**
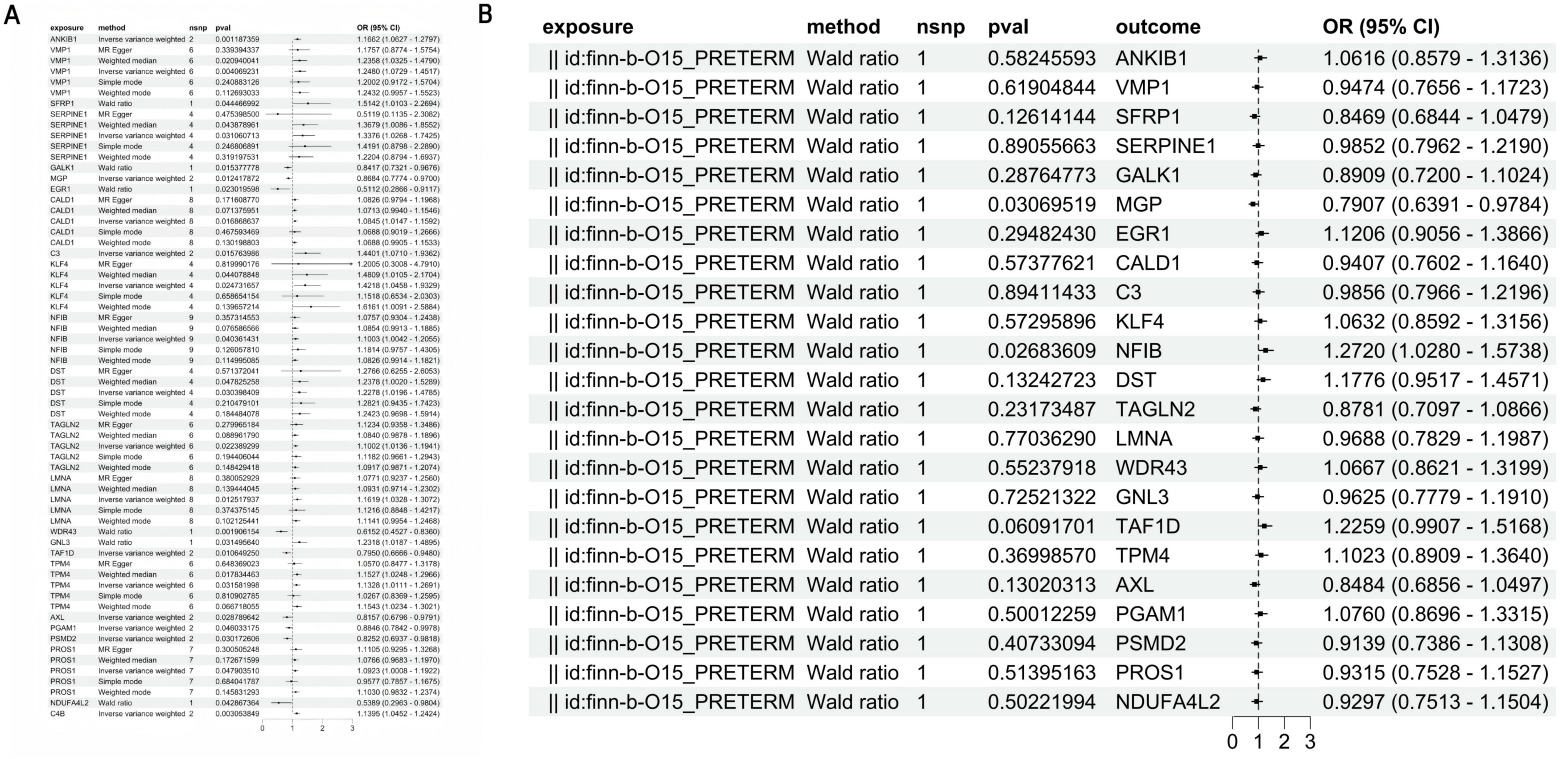
Mendelian randomization identifies KLF4 as a genetic risk factor for preterm birth. **(A)** KLF4 demonstrates a significant positive association with outcomes. **(B)** KLF4 did not reveal a significant negative association with the outcomes.

**FIGURE 5:**
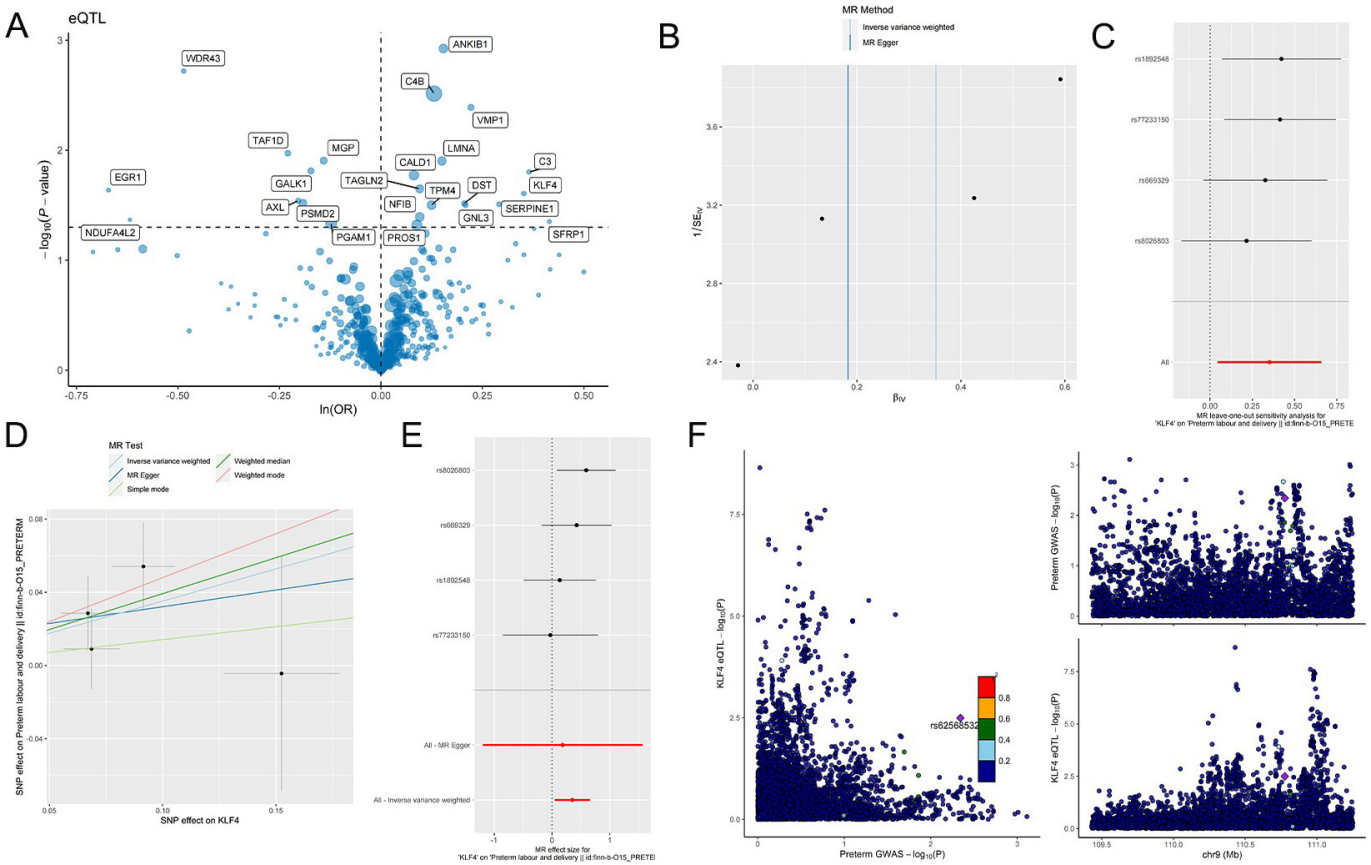
Mendelian randomization identifies KLF4 as a key preterm-associated molecule. **(A)** KLF4 is a genetic risk factor for preterm birth. **(B–E)** Analysis of KLF4-associated SNPs in eQTL datasets: **(B)** The effect estimates for KLF4 from the two Mendelian randomization approaches diverge to some extent. **(C)** Sensitivity analysis results for the four specific KLF4 SNPs (rs1092548, rs75723150, rs6695329, and rs9826033). **(D)** The SNP exerts a substantial effect on KLF4 and also has a significant impact on preterm birth. **(E)** SNPs in KLF4 have a significant effect on preterm birth. **(F)** GWAS and eQTL results provide evidence for a mechanism whereby specific SNPs contribute to preterm birth risk by modulating KLF4 expression.

We identified differentially expressed genes (DEGs) between the Ccl2+fib subpopulation and other fibroblast subpopulations (Atp8a1+Fib, Lum+Fib, Fos+Fib, Prap1+Fib, Il1b+Fib) as well as non-fibroblast cells from the initial clustering. Expression quantitative trait loci (eQTLs) of these marker genes were extracted from the IEU and Finnish GWAS databases as instrumental variables (IVs) (*p* < 5e-08). Strongly associated IVs were further selected based on an FDR > 10. Using these IVs, outcome variables were retrieved. Mendelian randomization (MR) analysis was subsequently performed, with results summarized in Figure 4A. To exclude reverse causality between IVs and outcomes, reverse MR analysis was conducted. The results demonstrated that most IVs were not influenced by reverse causation during preterm birth progression (Figure 4B). Visualization of the genetic effects of IVs on outcomes revealed that ANKIB1, C4B, VMP1, C3, KLF4, and SFRP were genetically identified as risk factors for preterm birth, whereas WDR43, EGR1, TAF1D, and MGP exhibited protective effects (Figure 5A). Krüppel-like transcription factors are critical for maintaining cellular functions, and their deficiency often leads to embryonic developmental abnormalities or lethality, underscoring their essential roles in embryogenesis. Therefore, KLF4 was selected as the target gene. MR analysis of KLF4 indicated a robust positive causal relationship with preterm birth (Figures 5B–E). Regional association plot analysis of KLF4 further confirmed its strong locus-specific genetic association (Figure 5F).

### KLF4 is consistently elevated in peripheral blood and placental tissues of preterm birth patients

**FIGURE 6:**
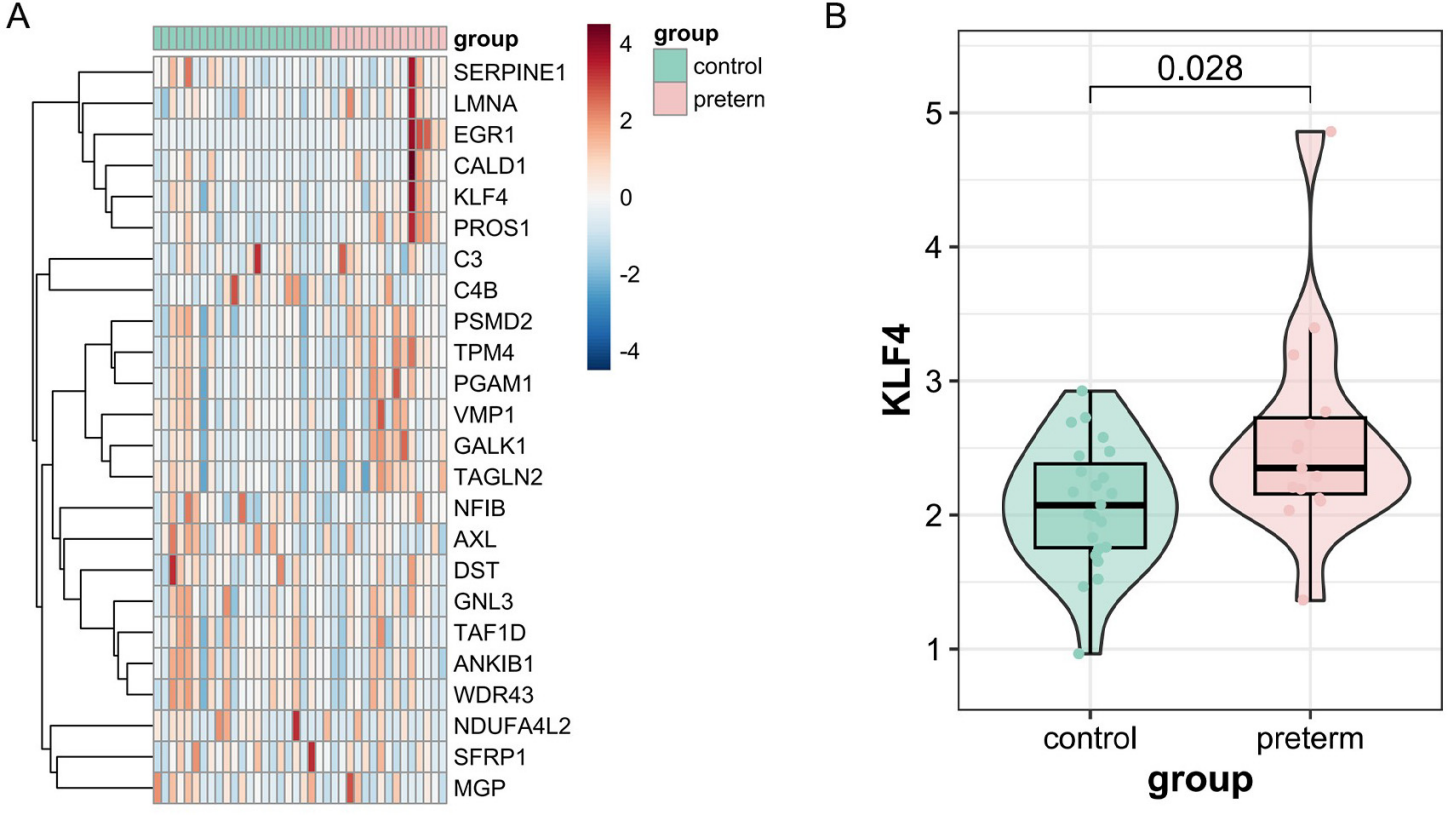
Elevated KLF4 expression in preterm patient blood and placenta. **(A)** Bulk-seq analysis of key genes (SERPINE1, LMNA, EGR1, CALD1, KLF4, PROS1) showing upregulated expression in preterm peripheral blood. **(B)** Significant KLF4 overexpression in preterm whole blood.

Analysis of the GSE96083 dataset revealed differential expression of key genes in peripheral blood between healthy controls and preterm birth groups. Heatmap results demonstrated that SERPINE1, LMNA, EGR1, CALD1, KLF4, and PROS1 were significantly upregulated in whole blood of preterm patients, consistent with prior MR findings (Figure 6A). Independent analysis of KLF4 further confirmed its elevated expression in preterm blood samples (Figure 6B). Additionally, placental tissues from 84 pregnant women were analyzed, showing that KLF4 expression was significantly higher in the preterm group compared to the term delivery group (Table 1).

**TABLE 1:** Comparison of the situation between full-term and preterm mothers.

Characteristics	Group	Full-term delivery (*n* = 50, %)	Preterm delivery (*n* = 34, %)	X^2^	*p*-value
Age		30.79 ± 5.83	30.5 ± 7.80	−1.581	0.118
Number of fetuses	2	13 (26.0)	8 (23.5)	0	1
	1	37 (74.0)	26 (76.5)		
Gravidity	2	15 (30.0)	14 (41.2)	0.679	0.41
	1	35 (70.0)	20 (58.8)		
Parity	2	22 (44.0)	14 (41.2)	0.001	0.974
	1	28 (56.0)	20 (58.8)		
Number of abortions	2	4 (8.0)	5 (14.7)	0.963	0.618
	1	10 (20.0)	6 (17.6)		
	0	36 (72.0)	23 (67.6)		
KLF4	High	44 (88.0)	23 (67.6)	4.009	0.045
	Low	6 (12.0)	11 (32.4)		
Scarred uterus	No	32 (64.0)	14 (41.2)	3.384	0.066
	Yes	18 (36.0)	20 (58.8)		
IVF-ET pregnancy	No	39 (78.0)	20 (58.8)	2.702	0.1
	Yes	11 (22.0)	14 (41.2)		
Anemia	No	31 (62.0)	13 (38.2)	3.679	0.055
	Yes	19 (38.0)	21 (61.8)		

### KLF4 knockdown attenuates LPS-induced placental inflammation

**FIGURE 7:**
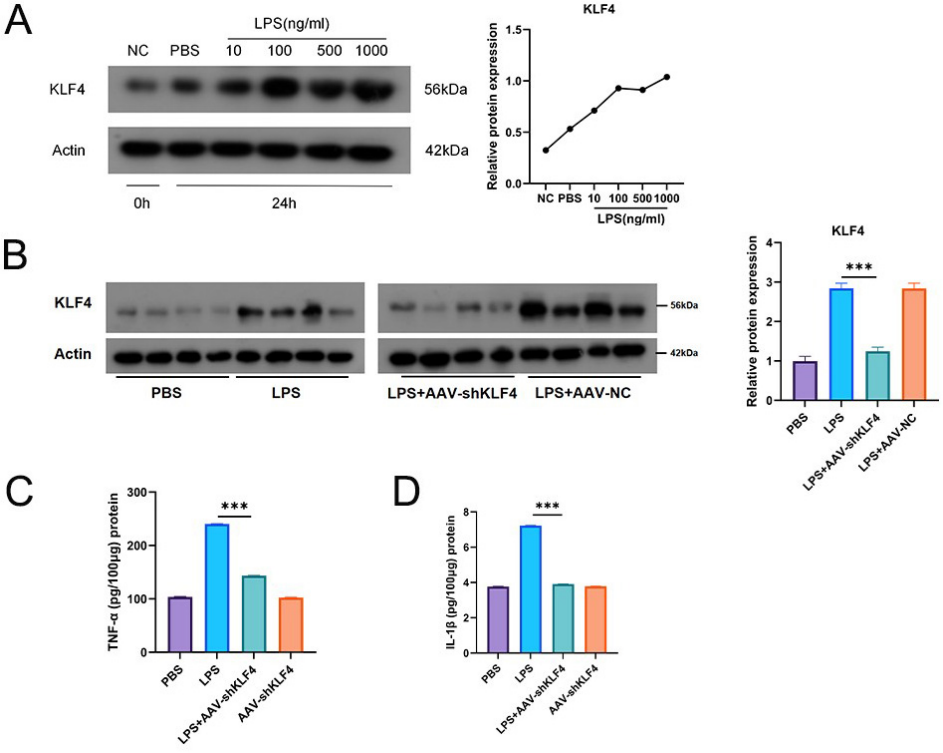
KLF4 underexpression alleviates placental inflammation. **(A)** Dose-dependent upregulation of KLF4 protein levels with increasing LPS stimulation. **(B)** KLF4 knockdown reverses LPS-induced KLF4 upregulation. **(C)** KLF4 knockdown suppresses LPS-induced secretion of TNF-α. **(D)** KLF4 knockdown suppresses LPS-induced secretion of IL-1β. ***P < 0.001.

To investigate the direct effects of KLF4 on placental tissue, we established a placental explant model. Placental explants were stimulated with gradient concentrations of lipopolysaccharide (LPS: 10, 100, 500, and 1,000 ng/mL) for 24 h. KLF4 protein expression exhibited a significant dose-dependent upregulation with increasing LPS concentrations (Figure 7A). To mimic an inflammatory microenvironment, isolated placental tissues were incubated with LPS (500 ng/mL) for 1 h, followed by co-culture with AAV-shKLF4 for an additional 24 h. Results demonstrated that KLF4 protein levels in the LPS group were significantly higher than those in the PBS control group. In contrast, the LPS+AAV-shKLF4 group showed a marked reduction in KLF4 expression compared to the LPS group. No significant difference in KLF4 levels was observed between the AAV-shKLF4 only group and the PBS control. Notably, KLF4 knockdown effectively alleviated LPS-induced cytotoxicity in placental cells (Figure 7B) and significantly suppressed LPS-triggered secretion of the pro-inflammatory cytokines TNF-α and IL-1β (Figures 7C, D).

### KLF4 knockdown alleviates intrauterine inflammation–induced preterm birth and fetal injury

**FIGURE 8:**
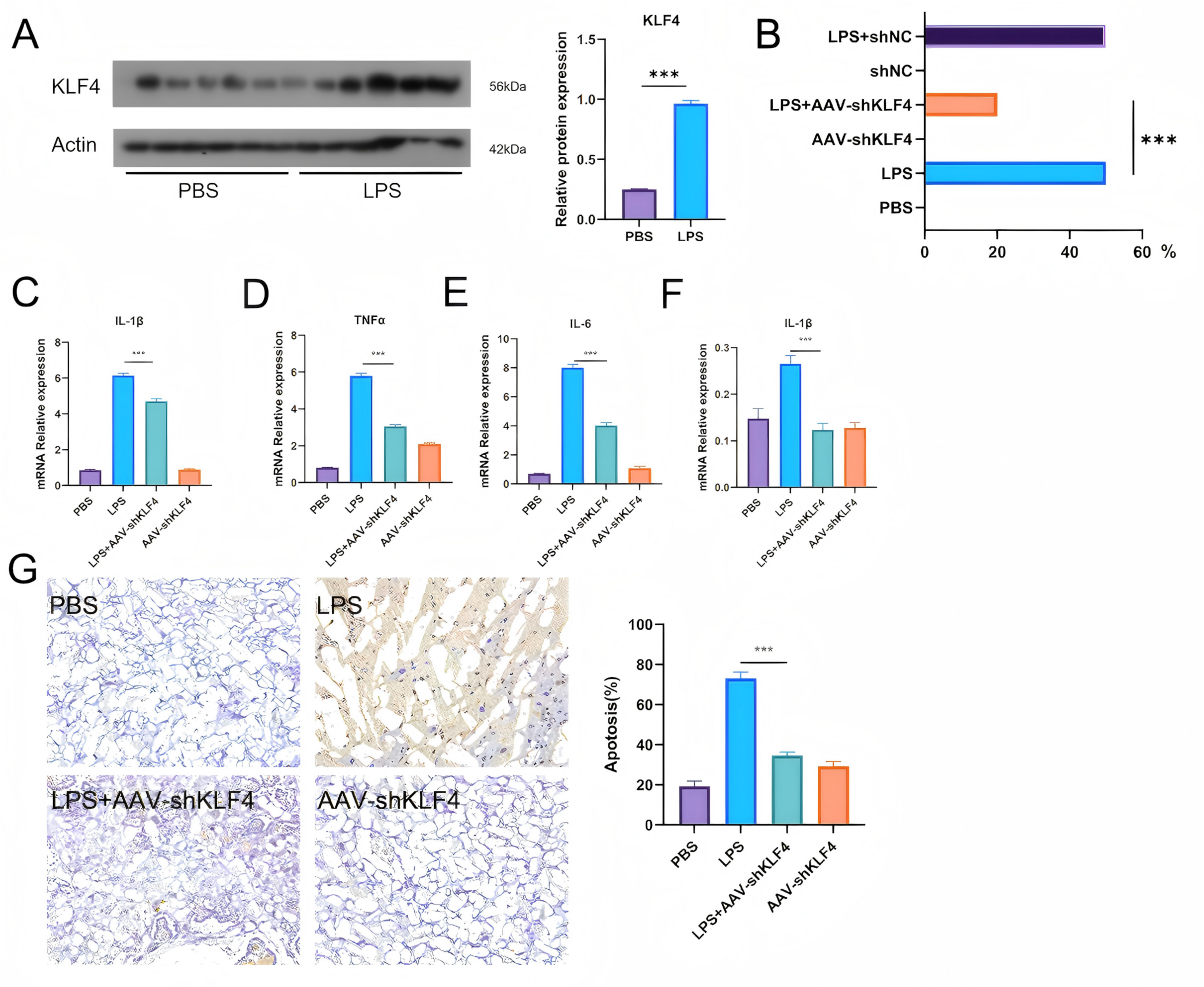
KLF4 underexpression mitigates intrauterine inflammation-induced preterm birth. **(A)** Protein expression of KLF4 in placental tissues was significantly increased in rats with intrauterine infectious inflammation. **(B)** KLF4 knockdown significantly reduced the incidence of fetal stillbirth in rats with intrauterine infectious inflammation. **(C)** KLF4 knockdown markedly decreased the mRNA expression level of IL-1β in the placenta of rats with intrauterine infectious inflammation. **(D)** KLF4 knockdown significantly downregulated the mRNA expression level of TNF-α in the placenta of rats with intrauterine infectious inflammation. **(E)** KLF4 knockdown led to a notable reduction in the mRNA expression level of IL-6 in the placenta of rats with intrauterine infectious inflammation. **(F)** KLF4 knockdown significantly suppressed IL-1β protein expression in the placenta of rats with intrauterine infectious inflammation. **(G)** KLF4 knockdown markedly reduced the number of apoptotic cells in the placenta of rats with intrauterine infectious inflammation. ***P < 0.001.

To further validate the association between *KLF4* and LPS-driven inflammatory responses, we established a rat model of intrauterine infection-induced inflammation using LPS. Results showed that placental KLF4 protein expression was significantly elevated in the LPS-induced intrauterine inflammation group compared to the PBS control group, suggesting its potential role as an inflammatory biomarker in the placenta (Figure 8A). Following treatment with AAV-shKLF4 via tail vein injection, the LPS-induced preterm birth rate was significantly reduced to 20%, accompanied by a marked decrease in fetal stillbirths (Figure 8B). Similarly, *KLF4* knockdown (LPS+AV-shKLF4 group) significantly suppressed placental mRNA levels of IL-1β, TNF-α, and IL-6 (Figures 8C–E). ELISA further confirmed that IL-1β protein expression in the LPS group was significantly higher than in the PBS group, whereas *KLF4* knockdown markedly reduced placental IL-1β protein levels (Figure 8F). Additionally, the number of apoptotic cells was significantly decreased in the LPS+AAV-shKLF4 group compared to the LPS group (Figure 8G).

## Discussion

Preterm birth (PTB) remains a leading cause of neonatal morbidity and mortality, yet its underlying biology—particularly for inflammation-associated PTB—remains incompletely understood (18, 19). In this study, we integrate single-cell transcriptomics, genetic causal inference, clinical observations, and functional validation to propose a fibroblast-centered inflammatory framework for PTB. Our data suggest that a Ccl2^+^ fibroblast subpopulation expands in PTB and functions as a prominent communication hub, while Mendelian randomization (MR) and experimental perturbation jointly support KLF4 as a key regulator that may amplify inflammatory signaling and contribute to adverse pregnancy outcomes.

A central observation from the single-cell atlas is the pronounced heterogeneity of fibroblasts at the maternal–fetal interface and the emergence of discrete fibroblast states with distinct functional programs. Among these states, the Ccl2^+^ fibroblast population is expanded in the PTB condition and displays enhanced signaling activity in cell–cell communication analyses. This finding is consistent with the broader concept of immune–stromal crosstalk, wherein stromal cells act not merely as structural components but as active organizers of inflammatory circuits (20). In the context of PTB, elevated CCL2 expression provides a plausible link to macrophage recruitment and amplification of local inflammation. Notably, the Ccl2^+^ fibroblast state may represent a convergent inflammatory program shared across tissues (21, 22). Therefore, the key contribution of our work is not the existence of inflammatory fibroblasts *per se*, but the placement of this fibroblast state within a PTB-relevant cellular and genetic framework.

To move beyond descriptive associations, we incorporated MR to prioritize candidate regulators with supportive genetic evidence. Using eQTL-based instruments, MR analysis implicates KLF4 as a risk-associated factor for PTB, and reverse MR analyses do not support reverse causality in our datasets (23, 24). Importantly, we interpret MR here as complementary evidence that strengthens prioritization rather than as a stand-alone demonstration of mechanism. As with any MR framework, residual pleiotropy or context-dependent genetic effects cannot be fully excluded (25, 26). Accordingly, we emphasize that genetic inference is most compelling when aligned with orthogonal functional data—an approach we adopted in subsequent experiments.

Consistent with the MR signal, KLF4 expression is elevated in peripheral blood transcriptomic data and in placental tissues from PTB patients, supporting clinical relevance. While these observations raise the possibility that KLF4 could contribute to PTB risk stratification, the present study is not designed to establish diagnostic performance. Prospective, multicenter cohorts with standardized sampling time points and adjustment for known confounders will be required to determine whether KLF4 provides incremental predictive value beyond established clinical risk factors.

Functional experiments further support a role for KLF4 in inflammation-associated PTB. In placental explants, inflammatory stimulation induces a dose-dependent increase in KLF4, and KLF4 knockdown attenuates pro-inflammatory cytokine production and reduces apoptosis-related readouts. In a rat intrauterine inflammation model, KLF4 knockdown markedly reduces LPS-associated PTB incidence and fetal loss, accompanied by decreased placental inflammatory markers. Together, these results suggest that KLF4 is not merely a biomarker of inflammation but may participate in reinforcing inflammatory and stress-response programs that are relevant to PTB pathophysiology.

Several limitations should be acknowledged. First, model generalizability: LPS-induced inflammation captures key aspects of infection-associated PTB but may not represent the full spectrum of spontaneous PTB etiologies. Second, cross-species inference: while KLF4 is conserved, differences between rodent models and human pregnancy may affect the translation of specific cellular programs. Third, cell-type specificity: systemic AAV-mediated knockdown may not isolate fibroblast-intrinsic effects, and future studies using cell-type–restricted perturbation (e.g., conditional knockdown/knockout or targeted delivery) will be important to dissect compartment-specific contributions. Finally, while single-cell analyses nominate the Ccl2^+^ fibroblast state and its communication patterns, spatial context is not directly captured; spatial transcriptomics or multiplex imaging will be valuable to confirm localization and interaction partners *in situ*.

In summary, our integrative approach supports a model in which expansion of a Ccl2^+^ fibroblast inflammatory state and a KLF4-associated regulatory program may contribute to inflammation-driven PTB. By connecting single-cell resolution, genetic causal inference, clinical observations, and functional perturbation, this study provides a coherent framework for understanding stromal–immune inflammatory networks at the maternal–fetal interface and motivates future work to define cell-type–specific mechanisms and evaluate translational potential in prospective human cohorts.

## Conclusion

In summary, this study integrates single-cell profiling, genetic inference, and functional perturbation to suggest that an expanded Ccl2^+^ fibroblast state contributes to inflammation-associated preterm birth and to implicate KLF4 as a regulatory factor linked to this stromal inflammatory program. Consistent with this framework, KLF4 knockdown attenuated placental inflammatory readouts and reduced inflammation-induced adverse pregnancy outcomes in our experimental models. Moving forward, priority should be given to defining KLF4 downstream targets and pathways in a cell-type–specific manner, validating the spatial architecture of fibroblast–immune interactions at the maternal–fetal interface, and testing clinical relevance in well-phenotyped prospective cohorts with PTB subtype stratification. These efforts will help clarify causal mechanisms and inform translational strategies aimed at mitigating inflammation-driven PTB.

## Data Availability

The original contributions presented in the study are publicly available. This data can be found here: GSE200289 (GEO) and E-MTAB-11491 (ArrayExpress).

## Supplementary material

SUPPLEMENTARY TABLE 1 Full ranked list of significant ligand–receptor pairs.
